# Epidemiology and Comorbidities of Excoriation Disorder: A Retrospective Case-Control Study

**DOI:** 10.3390/jcm9092703

**Published:** 2020-08-21

**Authors:** Christina Kwon, Nishadh Sutaria, Raveena Khanna, Erik Almazan, Kyle Williams, Noori Kim, Sarina Elmariah, Shawn G. Kwatra

**Affiliations:** 1 Department of Dermatology, Johns Hopkins University School of Medicine, Baltimore, MD 21205, USA; ckwon@jhmi.edu (C.K.); nishadh.sutaria@tufts.edu (N.S.); rkhanna8@jhmi.edu (R.K.); erikalmazan@jhmi.edu (E.A.); kwill223@jhmi.edu (K.W.); Nkim34@jhmi.edu (N.K.); 2 Department of Dermatology, Massachusetts General Hospital, Boston, MA 02114, USA; SBELMARIAH@mgh.harvard.edu

**Keywords:** excoriation disorder, skin-picking, itch, pruritus, epidemiology, comorbidities

## Abstract

Excoriation disorder is a psychocutaneous disorder characterized by repetitive skin-picking and associated with significant morbidity. Currently, epidemiological data in patients with excoriation disorder are lacking so we sought to characterize common patient demographics and comorbidities. We conducted a retrospective case-control study comparing 250 patients with excoriation disorder with 250 age-, race- and sex-matched controls identified between 2007 and 2019 at a single tertiary care center. We found that the majority of excoriation disorder patients were female (76%), Caucasian (82%) and unmarried (62%), with a mean age of 49 years. Compared to the matched controls, patients with excoriation disorder had increased odds of several psychiatric illnesses, including obsessive compulsive disorder (odds ratio (OR) 28.48, 95% confidence interval (CI): 1.68, 481.75), substance use disorder (OR 24.33, 95% CI: 5.81, 101.77), post-traumatic stress disorder (OR 8.23, 95% CI: 2.24, 129.40), depression (OR 8.19, 95% CI: 4.86, 13.80), bipolar disorder (OR 7.55, 95% CI: 2.22, 25.65), attention-deficit/hyperactivity disorder (OR 5.63, 95% CI: 1.62, 19.57), and anxiety (OR 5.01, 95% CI: 2.92, 8.62). Only a minority (42%) of patients were given psychiatry referrals and of those referred, a majority (64%) did not follow-up with psychiatry. The outcomes were also generally unfavorable as only 21% of patients experienced a resolution or improvement in their symptoms. This highlights the need for a multidisciplinary approach to manage patients with excoriation disorder, involving both dermatologists and psychiatrists.

## 1. Introduction

Excoriation disorder (ED, also known as psychogenic excoriations, neurotic excoriations, acne excoriée, pathological or compulsive skin picking, dermatotillomania, and body-focused repetitive behaviors) are characterized by excessive scratching or picking of the skin in the absence of an identified primary dermatosis, leading to secondary skin lesions and significant psychosocial distress [1,2,3,4]. ED has an estimated prevalence of 1.4–5.4% in the United States and is often associated with increased rates of psychiatric comorbidity [5,6,7,8]. The distribution of ED has been observed to be trimodal, with peaks occurring in childhood, adolescence to young adulthood, and middle adulthood [9]. The method of skin manipulation is highly variable, ranging from simply scratching to deliberate self-injurious behavior, resulting in a similarly highly variable presentation of lesions [10]. The Diagnostic and Statistical Manual of Mental Disorders, Fifth Edition (DSM-5) lists skin picking disorders as related to obsessive compulsive disorder (OCD) [11]. Patients with ED often go to dermatologists so importance must be placed on a better understanding of associated diseases and the presentation of ED. To date, few studies address the epidemiology, health comorbidities, and clinical outcomes of patients with ED, posing challenges to the diagnosis and treatment of ED [2,5,12,13]. While some studies have characterized different psychiatric illnesses associated with ED, the data have been limited by small sample sizes. Additionally, it has been suggested that many dermatologists are unfamiliar with the diagnosis and management of psychocutaneous disorders [14]. We aim to fill these knowledge gaps by characterizing the demographics, clinical characteristics, comorbidities, and outcomes for patients with excoriation disorder in this retrospective case-control study.

## 2. Experimental Section

We obtained institutional review board approval for the study (Johns Hopkins Medicine IRB; approval #00205377). A retrospective chart review was performed for patients seen by any provider at Johns Hopkins Hospital with the International Classification of Disease Ninth/Tenth Revision, Clinical Modification (ICD-9/10-CM) code L98.1 (factitial dermatitis) from 1 January 2007 to 1 May 2019, using the electronic medical record EPIC. Patients were deemed to have excoriation disorder if they had a diagnosis of “neurotic excoriations,” “skin-picking” or “excoriation disorder”; these diagnoses were considered synonymous for the purpose of this study. For these patients, data regarding their demographics, types and duration of symptoms, physical exam findings, systemic and psychiatric comorbidities, clinical course, treatment, psychiatric medications, and outcomes were collected. The outcomes were defined as a complete resolution of the disease, improvement of the disease, no change in the disease, or worsening of the disease as determined by the patient and physician’s impressions. To identify the health comorbidities affecting this population, ED patients were compared to age-, race-, and sex-matched controls in a 1:1 ratio. The control cases were randomly selected from lists of patients seen by a single provider at the dermatology clinic for benign conditions, including benign melanocytic nevi, acne vulgaris, and alopecia areata.

Descriptive statistics were calculated using Microsoft Excel 2017. Further statistical analyses to compare the ED and control cohorts and calculate odds ratios (OR) and 95% confidence intervals (CI) were performed with JMP v. 12.1.0 (SAS Institute, Cary, NC, USA). The continuous variables were analyzed with a Student’s *t*-test, while the categorical variables were analyzed by the chi-squared or Fischer’s exact test. The statistical significance was determined with a cutoff of *p* < 0.05. The Haldane–Anscombe correction was applied to groups with zero patients to calculate odds ratios [15]. The Benjamini–Hochberg method was used to adjust the *p*-values for multiple tests (*k* = 20) [16].

## 3. Results

In total, 315 adult (age ≥ 18 years) patients were identified with the ICD-9/10-CM code L98.1, of which 250 patients (79%) had a diagnosis of excoriation disorder. Of the identified ED cases, 190 (76%) were female and 60 were male (24%) (Table 1). There were 204 (82%) Caucasians, 34 (14%) Blacks, 7 (3%) Others, and 5 (2%) Asians. The average age at diagnosis was 49 ± 16 years and the average reported duration of symptoms at the time of diagnosis was 2.9 ± 0.4 years. In terms of employment, 119 (48%) were unemployed. The majority of ED patients were single, divorced, widowed or legally separated (n = 154, 62%).

### 3.1. Comorbidities Associated with Excoriation Disorder

**Figure 1:**
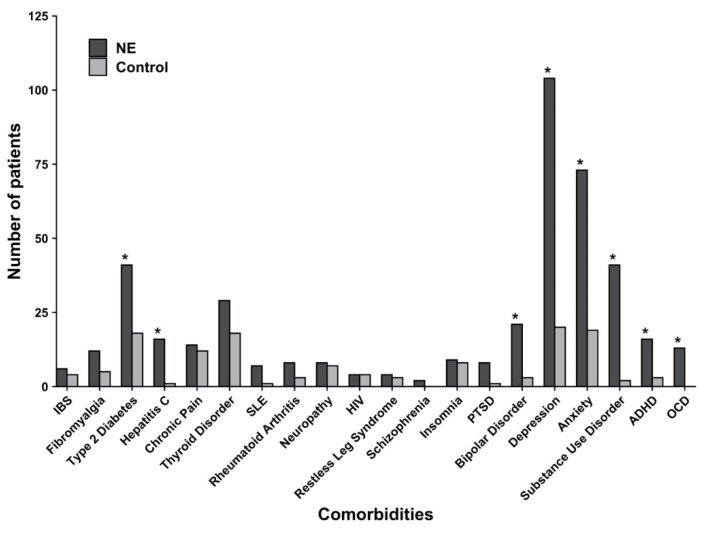
Number of patients with queried comorbidities in the ED and control groups. * Denotes statistically significant differences as determined by a Student’s *t*-test (*p* < 0.05). ED: excoriation disorder; IBS: irritable bowel syndrome; SLE: systemic lupus erythematosus; HIV: human immunodeficiency virus; PTSD: post-traumatic stress disorder, ADHD: attention-deficit/hyperactivity disorder; OCD: obsessive compulsive disorder.

Systemic comorbidities were observed in a subset of ED patients, with type 2 diabetes mellitus (n = 41, 16%), thyroid dysfunction (n = 29, 12%) and hepatitis C (n = 16, 6%) being the most common (Figure 1). The majority of ED patients (n = 185, 74%) had at least one comorbid psychiatric disorder. The most common psychiatric comorbidities encoded were depression (n = 104, 42%), anxiety (n = 73, 29%) and substance use disorder (n = 41, 16%). Relative to the matched controls, ED was associated with increased odds of concomitant OCD (OR 28.48, 95% CI: 1.68, 481.75), substance use disorder (OR 24.33, 95% CI: 5.81, 101.77), hepatitis C (OR 17.03, 95% CI: 2.24, 129.40), depression (OR 8.19, 95% CI: 4.86, 13.80), bipolar disorder (OR 7.55, 95% CI: 2.22, 25.65), attention-deficit/hyperactivity disorder (ADHD; OR 5.63, 95% CI: 1.62, 19.57), anxiety (OR 5.01, 95% CI: 2.92, 8.62), and type 2 diabetes (OR 2.53, 95% CI: 1.41, 4.54) (Table 2).

### 3.2. Clinical Features and Treatment Patterns of Excoriation Disorder

Most patients (n = 176, 71%) complained of a pruritic (“itchy”) sensation symptoms. The most common sites of ED were the upper extremities (n = 158, 63%), trunk (n = 147, 59%), lower extremities (n = 128, 51%), face (n = 112, 45%) and scalp (n = 22, 9%) (Table 3). The most common complications were ulceration (n = 49, 20%) and infection (n = 23, 9%). Only 59 (24%) patients received an additional work-up such as cultures, biopsies, thyroid studies, serum/urine protein electrophoresis (SPEP/UPEP), lactate dehydrogenase (LDH), complete blood count (CBC), comprehensive metabolic panel (CMP), and hepatitis serologies. No underlying malignancies were identified.

At the time of ED diagnosis, most patients (n = 178, 71%) were taking at least one psychiatric medication. Only 59 (24%) patients reported a history of regular follow-ups with a psychiatric or mental health service provider. The most common psychiatric medications prescribed for patients with ED included selective serotonin reuptake inhibitors (SSRIs) (n = 117, 27%), benzodiazepines (n = 51, 20.4%), and heterocyclic antidepressants (n = 50, 20%). Antibiotics (topical and/or oral), antihistamines and topical corticosteroids were initiated for the treatment of ED in 142 (57%), 57 (23%) and 131 (52%) cases, respectively. Other therapies such as doxepin (n = 42, 17%), narrowband ultraviolet-B (UV-B) (n = 13, 5%), N-acetylcysteine (n = 8, 3%), camphor-menthol (n = 19, 8%) and olanzapine (n = 3, 1%) were also used. Anti-parasite treatments, such as ivermectin and permethrin, were infrequently administered (n = 9, 4%).

### 3.3. Outcomes of Excoriation Disorder

The average number of dermatology return visits by ED patients in the specified date range (1 January 2009 to 1 May 2019) was 2.0 ± 0.3. A minority of ED patients (n = 113, 45%) were given referrals to psychiatry and only 36% (n = 41) of those given referrals had documented follow-ups with a psychiatric or mental health provider. The prognoses were highly variable with only 11 (4%) patients experiencing resolution, 42 (17%) improved, 5 (2%) worsened, 91 (34%) experienced no change and 106 (42%) were lost to follow-up (Table 1).

## 4. Discussion

In this study, we found that patients with ED are predominately female, Caucasian and unmarried. Patients often reported an itch as a symptom associated with their picking behaviors. The high representation of females may be partly explained by a diagnostic bias among providers. Studies have shown that medical complaints by female patients are more likely to be downplayed and labeled psychosomatic compared to those of male patients, leading to a delay in treatment and worse patient outcomes [17,18]. It is therefore possible that females presenting pruritus are more likely to be diagnosed with ED as opposed to an itch due to a secondary cause. A false diagnosis of ED could then lead to an inadequate work-up for the underlying causes of the presenting itch. Though further research is needed to validate this hypothesis, providers must be aware of their own implicit biases when diagnosing ED to avoid delaying necessary treatments.

The clinical assessment of ED involves a thorough history to screen for psychiatric disease and a full dermatologic exam with focus particularly on the face and arms [10]. Indeed, we found that the upper extremities and face were among the most affected regions, along with the trunk and lower extremities. Questionnaire-based instruments such as the Skin Picking Severity Scale (SPS) and Skin Picking Impact Scale (SPS) can also be helpful to quantify disease severity [10]. Additionally, not all patients reported pruritus as a symptom, suggesting that some patients have a premonitory urge to pick at their skin without an itch as a driving motivator. This dichotomous pattern has been observed in trichotillomania as well as ED in prior studies [19,20]. Further investigation of the motivations behind ED may explain ED’s relationship with psychiatric disease.

Compared to matched controls, ED patients in this study were more likely to have type 2 diabetes and hepatitis C, two conditions which have previously been reported to be associated with chronic pruritus [21]. To our knowledge, associations between ED and these systemic conditions have not yet been reported. Although the significance of this observation remains unclear, the increased frequencies of diabetes and hepatitis C in ED patients are unlikely to account for the frequency of pruritus observed in our sample. Our patients also had a high prevalence of comorbid psychiatric disease, with 74% of patients having at least one psychiatric comorbidity. Depression was the most commonly associated disorder in our ED population, consistent with a prior report examining the psychiatric profiles of 50 patients with ED [2]. Following depression, the most common psychiatric comorbidities observed were anxiety, substance use disorder and bipolar disorder. Less than 8% of patients were diagnosed with either ADHD, OCD, insomnia, schizophrenia or restless leg syndrome. It is possible that a diagnostic bias resulted in an overestimation of odds, as patients may be more likely to receive a diagnosis of ED if they already have a preexisting psychiatric illness. Nevertheless, patients with ED overall demonstrated increased odds of most of the psychiatric illnesses analyzed compared to matched controls, with the highest odds being observed in patients with substance use disorder and OCD.

The high burden of psychiatric disorders observed in our study is consistent with prior reports suggesting a close relationship between ED and psychiatric disease [1]. It has been proposed that ED should fall under the label “self-inflicted skin lesion” (SISL) and that any self-mutilation arising as part of a mental disorder should preclude the diagnosis of ED [4]. This classification system may be too restrictive however since ED also shares features with many other psychiatric illnesses. Despite the imperative for a multidisciplinary approach involving dermatologic and psychiatric care in ED management, less than half of ED patients in our study were referred to psychiatry. Previous reports have demonstrated that only 42% of dermatologists feel very comfortable diagnosing and managing psychocutaneous disorders and 90% of dermatologists are unaware of any patient resources, underscoring the need for dermatologist education and co-management with psychiatrists [13,22]. The Association of Psychoneurocutaneous Medicine of North America (APMNA) is one educational resource available to both providers and patients. Additionally, of those patients who were referred to psychiatry, only 36% had a documented visit with a psychiatrist or mental health provider. The reason for the low rate of successful psychiatry referrals in the ED population is unclear. One potential reason is that a large proportion of patients were already on antidepressants or other psychiatric medications at the time of ED diagnosis, leading to a false sense that their mental health needs were already being addressed. An average of two dermatology return appointments also suggests an inadequate continuity of care, however it is unclear if this finding was due to lack of scheduling by the provider or poor follow-up by patients. Nonetheless, the lack of multidisciplinary care for ED patients highlights an area in which much improvement is needed and underscores the important role dermatologists play in being a gatekeeper to the health care system for patients with ED.

In addition to inadequate psychiatric follow-ups, the lack of effective treatment options likely contributed to the poor patient outcomes in our study. Currently, there are no pharmacological treatments approved by the US Food and Drug Administration (FDA) for this condition and the pathophysiology of ED is unknown. Off-label therapy with SSRIs and lamotrigine has had variable success [23,24,25,26,27]. Recent functional magnetic resonance imaging (fMRI) studies have shown abnormal brain activation in patients with ED, suggesting that abnormal neural circuitry in habit formation, action monitoring and inhibition may be involved in the pathophysiology of ED [28,29]. Indeed, N-acetylcysteine therapy has been shown reduce skin-picking behaviors, presumably by improving the glutamatergic dysfunction implicated in the pathophysiology of compulsive behaviors [30]. Other pharmacotherapies with variable success for ED treatments include opioid antagonists, antipsychotics, and inositol [10,31]. Further research on the pathophysiology of ED is needed to elucidate more effective treatment options.

The limitations of this study include its retrospective nature and patient selection from a single, tertiary care center. The selection of both cases and controls from a tertiary center may have resulted in an overrepresentation of patients with severe, recalcitrant disease; such a sampling bias limits the generalizability of the findings. The odds ratios for some diseases (i.e., OCD and schizophrenia) may be imprecise due to a low sample size. Provider biases of over diagnosing ED in females and patients with existing psychiatric disease may have also occurred, which would limit the internal validity and cause an overestimation of the odds of psychiatric comorbidities.

## 5. Conclusions

In conclusion, the majority of our ED patients were female, Caucasian, unmarried and middle-aged. The most common systemic abnormality was underlying type 2 diabetes. We observed a high burden of psychiatric diseases, notably depression and anxiety. Only a minority of patients were referred to psychiatry and, of those who were referred, approximately one-third had a documented psychiatry visit. The outcomes were generally not favorable as few patients achieved a resolution or improvement in their symptoms. Currently there is still no well-established treatment or medication approved by the FDA for excoriation disorder. Further multicentered, prospective studies are needed to better understand the disease pathophysiology and effective treatment methods. Furthermore, a multidisciplinary approach is needed to provide optimal care for patients with excoriation disorder.

## Figures and Tables

**Table 1:** Demographics of 250 patients with excoriation disorder (ED).

Patient Characteristics	All Patients	Resolution of Disease, n (%)	Improvement of Disease, n (%)	No Change or Worsened, n (%)
Number of patients	250	11 (4)	42 (17)	91 (36)
Sex, n (%)				
Female	190 (76)	7 (4)	35 (18)	70 (37)
Male	60 (24)	4 (7)	7 (12)	21 (35)
Race, n (%)				
Caucasian	204 (82)	8 (4)	35 (17)	73 (36)
Black	34 (14)	2 (6)	6 (18)	15 (44)
Other	7 (3)	0	0	2 (2)
Asian	5 (2)	1 (9)	1 (2)	1 (1)
Marital Status, n (%)				
Single	110 (44)	7 (6)	14 (13)	40 (36)
Married	83 (33)	2 (2)	13 (16)	31 (37)
Divorced/Separated	29 (12)	2 (7)	7 (24)	10 (34)
Widowed	15 (6)	0 (0)	5 (33)	5 (33)
Employment Status, n (%)				
Unemployed	119 (48)	5 (4)	20 (17)	50 (42)
Disease Characteristics				
Duration (years), mean ± SD	2.9 ± 0.4 (n = 224)	3.1 ± 1.3 (n = 11, 5%)	5.4 ± 2.0 (n = 36, 16%)	1.7 ± 0.3 (n = 78, 35%)

ED: excoriation disorder; SD: standard deviation.

**Table 2:** Demographics and case-control analysis of the comorbidities of ED patients and age-, race-, and sex-matched controls.

Patient Characteristics/Diseases	Excoriation Disorder (n = 250)	Control (n = 250)	Odds Ratio (95% CI)	Adjusted *p*-Value
Age (years), mean ± SD	49 ± 16	49 ± 16	---	---
Sex, n (%)				
Male	60 (24)	60 (24)	---	---
Female	190 (76)	190 (76)	---	---
Race/Ethnicity, n (%)				
Caucasian	204 (82)	204 (82)	---	---
Black	34 (14)	34 (14)	---	---
Asian	5 (2)	5 (2)	---	---
Other	7 (3)	7 (3)	---	---
Medical Comorbidities, n (%)				
IBS	6 (2)	4 (2)	1.51 (0.42, 5.43)	0.939
Fibromyalgia	12 (5)	5 (2)	2.47 (0.86, 7.12)	0.228
Type 2 Diabetes	41 (16)	18 (7)	2.53 (1.41, 4.54)	0.006
Hepatitis C	16 (6)	1 (0.4)	17.03 (2.24, 129.40)	<0.001
Chronic Pain	14 (6)	12 (5)	1.18 (0.53, 2.60)	0.916
Thyroid Disorder	29 (12)	18 (7)	1.69 (0.91, 3.13)	0.167
SLE	7 (3)	1 (0.4)	7.17 (0.88, 58.73)	0.136
Rheumatoid Arthritis	8 (3)	3 (1)	2.72 (0.71, 10.38)	0.340
Neuropathy	8 (3)	7 (3)	1.15 (0.41, 3.21)	0.933
HIV	4 (2)	4 (2)	1 (0.25, 4.04)	1.000
Restless Leg Syndrome	4 (2)	3 (1)	1.34 (0.30, 6.04)	1.000
Psychiatric Comorbidities, n (%)				
Schizophrenia	2(1)	0 (0)	5.04 (0.24, 105.52) *	0.713
Insomnia	9 (4)	8 (3)	1.13 (0.43, 2.98)	1.000
PTSD	8 (3)	1 (0.4)	8.23 (1.02, 66.31)	0.082
Bipolar Disorder	21 (8)	3 (1)	7.55 (2.22, 25.65)	0.002
Depression	104 (42)	20 (8)	8.19 (4.86, 13.80)	0.002
Anxiety	73 (29)	19 (8)	5.01 (2.92, 8.62)	0.002
Substance Use Disorder	41 (16)	2 (1)	24.33 (5.81, 101.77)	<0.001
ADHD	16 (6)	3 (1)	5.63 (1.62, 19.57)	0.010
OCD	13 (5)	0 (0)	28.48 (1.68, 481.75) *	<0.001

* Denotes calculations for which the Haldane–Anscombe correction was applied to avoid dividing by zero. CI: confidence interval; SD: standard deviation; IBS: irritable bowel syndrome; SLE: systemic lupus erythematosus; HIV: human immunodeficiency virus; PTSD: post-traumatic stress disorder; ADHD: attention-deficit/hyperactivity disorder; OCD: obsessive compulsive disorder.

**Table 3:** Clinical features of ED.

Clinical Characteristics	Number of ED patients (%)
Reported pruritus	176 (71)
Lesion Location	
Scalp	22 (9)
Face	112 (45)
Trunk	147 (59)
Upper extremities	158 (63)
Lower extremities	128 (51)
Complications	
Ulceration	49 (20)
Infection	23 (9)
